# Short Lives with Long-Lasting Effects: Filopodia Protrusions in Neuronal Branching Morphogenesis

**DOI:** 10.1371/journal.pbio.1002241

**Published:** 2015-09-03

**Authors:** George Leondaritis, Britta Johanna Eickholt

**Affiliations:** 1 Department of Pharmacology, Medical School, University of Ioannina, Ioannina, Greece; 2 Institute of Biochemistry & Neuro Cure Cluster of Excellence, Charité – Universitätsmedizin Berlin, Berlin, Germany

## Abstract

The branching behaviors of both dendrites and axons are part of a neuronal maturation process initiated by the generation of small and transient membrane protrusions. These are highly dynamic, actin-enriched structures, collectively called filopodia, which can mature in neurons to form stable branches. Consequently, the generation of filopodia protrusions is crucial during the formation of neuronal circuits and involves the precise control of an interplay between the plasma membrane and actin dynamics. In this issue of *PLOS Biology*, Hou and colleagues identify a Ca^2+^/CaM-dependent molecular machinery in dendrites that ensures proper targeting of branch formation by activation of the actin nucleator Cobl.

## Abstract 2

A new study provides novel insight into how calcium signalling can control the branching of dendrites during nervous system development.

## Introduction

Thin, finger-like, dynamic membrane protrusions, filopodia, extending from the plasma membrane of cells were first described some 100 years ago and, since then, their definition is still largely based on morphological criteria. Structurally, classical filopodia are composed of unbranched, bundled actin filaments, which are polarized towards the tip of the filopodial membrane. As the actin filaments grow at their rapidly polymerizing barbed ends, protrusions of finger-like membrane domains occur. Whilst filopodia of different cell types or distinct specialized cellular compartments show a remarkably similar macroscopic appearance, they may nevertheless differ substantially in their ultrastructure, molecular composition, motility and functionality [1–4]. In the interest of simplicity, however, we would like to emphasize that we use here the term “filopodia” or “filopodia protrusions” throughout to collectively describe finger-like membrane protrusions as precursors of axon or dendrite branches, or dendritic spines.

### Dendritic Membrane Filopodia-like Protrusions as Precursors of Branches or Spines

In neurons, it has long been argued that filopodia originating from dendrite shafts are different to classical filopodia originating in the growth cones of dendrites and axons. For example, dendritic shaft filopodia are composed of actin filaments that are not as tightly bundled as those found in classical filopodia [2]. Dendrite filopodia also contain actin filaments of mixed polarity, which suggests that they can extend at the filopodia tip as well as at the filopodia root [5]. This further implies that actin-dependent motor proteins, like myosin II, can induce contractility of these structures [2]. As well as long actin filament bundles, dendritic filopodia contain branched actin filaments that derive from a specific set of actin nucleators. This characteristic specific to dendrites is thought to provide further structural plasticity to the nascent filopodium, which is essential for the transition of the filopodium into a dendritic spine or a dendrite branch [3,4].

A wealth of studies directly links the generation of filopodia-like protrusions to dendritic branching, arborization, and spine formation during morphogenesis and establishment of synaptic contacts in the developing brain [3,6]. Defects in these processes are believed to contribute to specific neurological diseases in the adult brain [7]. Considering the timing and control of these processes, both intrinsic and extrinsic signals are instrumental. Extrinsic signals such as neurotrophic factors, adhesion molecules, presynaptic neurotransmitter cues, and neuronal activity are particularly important, and all contribute to this highly dynamic process [6,8–10].

### How Are Filopodia Protrusions Formed?

**Fig 1:**
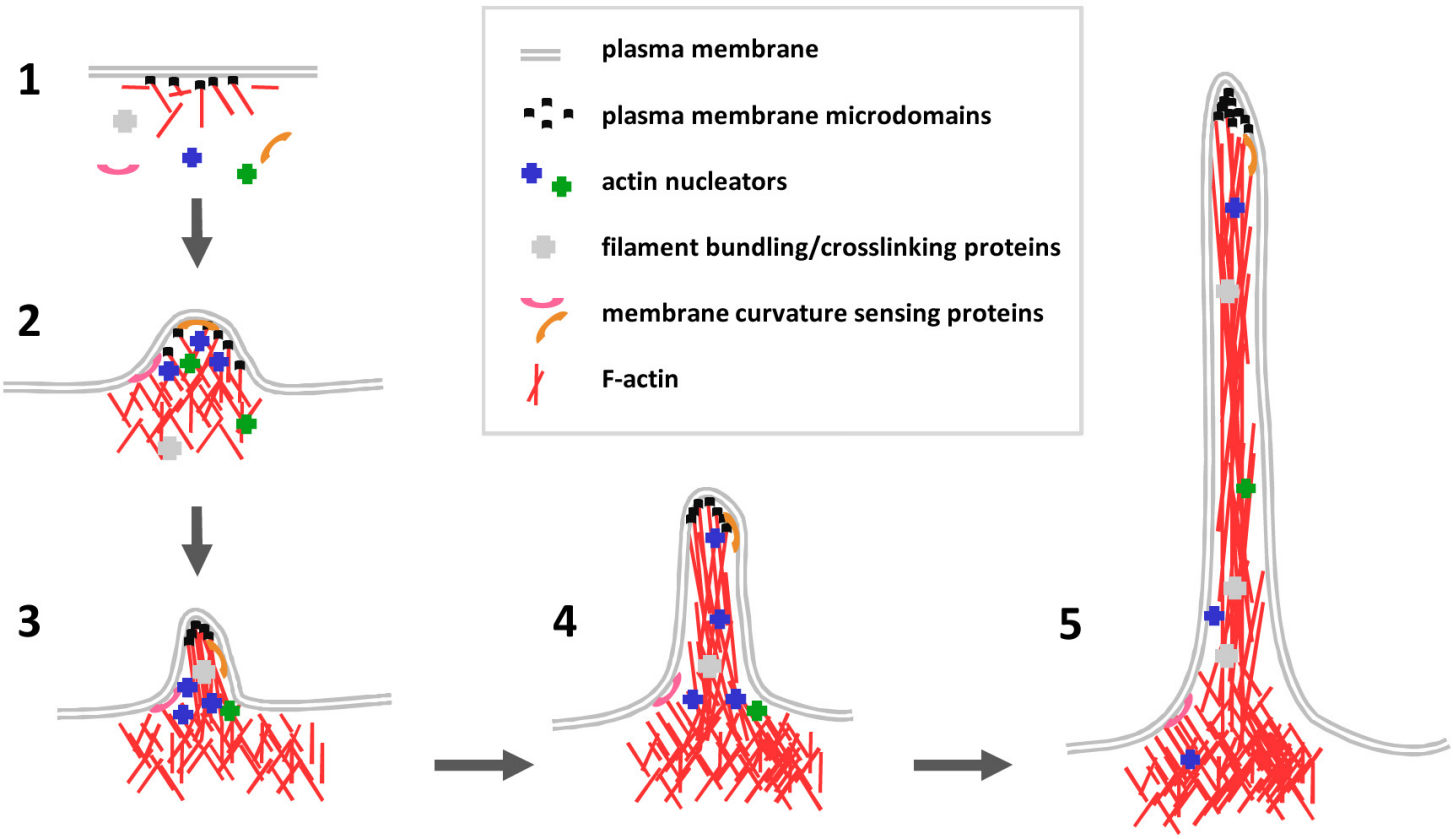
Filopodia formation in five steps. 1. Under resting conditions, actin nucleators (blue and green crosses), filament bundling and crosslinking proteins (grey crosses), and membrane curvature–sensing proteins (purple and orange curved lines) reside in the cytosol and preserve a base-line level of filamentous (F-) actin (red lines). Black dots denote plasma membrane microdomains rich in specific lipids and transmembrane proteins. 2. Upon signaling (e.g., increases in Ca^2+^ in the cytosol or activation of growth factor induced Phosphatidylinositol-4,5-bisphosphate (PIP2)-Phosphatidylinositol-3,4,5-trisphosphate (PIP3) turnover), nucleators are activated and, together with elongation factors, promote rapid actin polymerization and actin patch formation. 3. Filaments extend rapidly towards the membrane, and changes in membrane curvature are sensed and/or induced by curvature-sensing proteins. 4. A growing filopodium has established a mixture of unbundled and bundled or crosslinked actin filaments. It is conceivable that different sets of nucleators and elongators may contribute to increased actin polymerization. Other proteins that uncap or cap barbed ends or proteins that sever filaments at the root of filopodia may regulate filament turnover during this process. 5. In a “mature” filopodium, additional signals may guide microtubule invasion in order to stabilize the nascent filopodium into a branch.

Filopodia formation can be seen as a sequence of three interconnected phases: (a) the phase of actin nucleation, (b) the phase of rapid elongation of an actin filament through barbed-end polymerization, and (c) the bundling of actin filaments (Fig 1). An increasing list of filopodia regulators have been identified and characterized in recent years [3,11,12]. Among them, the actin nucleators take on specific importance during the formation of filopodia protrusions, and they do so for several reasons. For example, actin nucleation is the first, and rate-limiting, step in protrusion formation, and the control of actin nucleators provides an attractive mechanistic switch that can link external signals with filopodia production.

Actin nucleators are classified in three classes depending on their mode of action. Class I nucleators include the first nucleator identified, the Actin-related proteins 2/3 (Arp2/3) complex, and its accessory nucleation-promoting factors such as Neural Wiskott-Aldrich Syndrome Protein (N-WASP) and WASP-family Verprolin-homologous protein (WAVE-1). The regulation of Arp2/3 complex is intricate, but consensus has it that stimulation of the small GTPases Rac1 and Cdc42 by membrane the phosphoinositides PIP2 and PIP3 is an essential component of the Arp2/3 upstream activation machinery. Following filopodia formation, Arp2/3 then creates branched actin filaments and functions as the major actin nucleator responsible for the formation of lamellipodia at the leading edge of migrating cells and neuronal growth cones [13,14]. Class II nucleators include formins, such as Mammalian Diaphanous-related 1–3 (mDia1-3), which can function as elongators of actin filaments frequently found at the tip of a filopodium. Formins are recruited to the plasma membrane by interactions with acidic phospholipids and the GTPase Rif [12,15]. In the case of dendrite filopodia, for example, the formin mDia2 and Rif, in combination with Cdc42 and the Arp2/3 complex, act in a concerted manner during the transition from a nascent filopodium into a spine [3,5]. Class III nucleators include a set of newly identified diverse proteins such as the Cordon-Bleu WH2 repeat protein (Cobl), Spire, and Leiomodin, all of which share a common Wasp Homology 2 (WH2) domain to achieve nucleation [11,13,16]. To date, candidate upstream regulators or signals that induce the activity of these class III nucleators are not well described.

### A Novel Mechanism for Signal-Induced Formation of Actin Patches and Dendrite Branches

What is exciting about the contribution made by Hou et al. in *PLOS Biology* is that their paper delineates a novel regulatory mechanism for the formation of dendritic filopodia and dendrite arborization by class III nucleators [17]. The authors studied the class III actin nucleator Cobl, which had previously been identified as an important mediator of dendritic branch formation [16]. By using a variety of cellular and molecular approaches, the authors demonstrate that Cobl connects localized raises in Ca^2+^ in dendrites to the initiation of dendritic protrusion and branches. Live cell imaging reveals a correlation of local Ca^2+^ increases, Cobl accumulation, and F-actin patches at sites of dendrite branching. Interestingly, whilst increases in Ca^2+^ either preceded or followed the formation of actin patches, the maximum of Cobl accumulation appeared prior to the formation of actin patches at sites of extending filopodia protrusions. Cobl recruitment to the initiated protrusion required calmodulin (CaM), and live cell imaging indicated a timed correlation between local Ca^2+^ increases, CaM, and Cobl recruitment to sites along the dendritic membrane with somewhat protrusive morphology. Given that overexpression of Cobl has been shown to increase the number of dendrites and dendritic branches in hippocampal neurons [16], the authors went on to investigate if the activity of Ca^2+^/CaM is necessary for this phenotype. Indeed, CaM inhibitors suppressed Cobl-induced dendritic arborization and, furthermore, they produced a rapid shutdown of filopodia dynamics and gradual loss of Cobl from putative initiation sites. Conversely, Cobl mutants unable to bind to Ca^2+^/CaM failed to increase dendritic numbers and branches and were unable to rescue the phenotype of Cobl-silenced branching in cerebellar Purkinje neurons.

A crucial aspect of the initial phases of actin nucleation and filopodia initiation is the association of the regulators to the plasma membrane. In some cases, reorientation of actin filaments towards membranes by actin nucleators is ensured and guided by membrane phospholipids such as PIP2 or PIP3 [18,19]. In some instances, localized increases in actin dynamics and the formation of actin patches have been shown to correlate with accumulations of growth factor–induced PIP3 in membrane microdomains [4]. In a well-described model for initiation of axonal filopodia, for example, PIP3 microdomains provide a signal for recruitment of actin nucleators such as the Arp2/3 complex and its associated promoting factors and activating GTPases to the membrane. This sequence of events then increases the probability of generating an actin patch, which may develop into a membrane filopodium upon further expansion and elongation [20,21]. Alternatively, proteins that interact specifically with membranes, such as proteins of the F-Bin–Amphiphysin–Rvs (BAR) and I-BAR subfamilies involved in sensing membrane curvature, may control both actin nucleation activity of Cobl and membrane association. The authors probed for the effects of Ca^2+^ and Ca^2+^/CaM on known interactions of Cobl with actin and the F-BAR protein syndapin. Collectively, the results of these experiments demonstrate that local and transient raises in Ca^2+^ induce a net increase of both the actin-binding and syndapin-binding properties of Cobl. Cobl may be able to respond to transient, but not long-lasting, Ca^2+^/CaM signaling. This correlates well with previous observations that provided a link between local Ca^2+^ transients and filopodia outgrowth, whilst high levels of Ca^2+^ inhibited the formation of filopodia [22].

## Conclusion

The work by Hou et al. establishes Cobl΄s role as a Ca^2+^ sensor that can induce the initiation of filopodia-like protrusions on dendrites in a Ca^2+^/CaM-dependent manner. The question then arises what the source or the initial signal for this Ca^2+^ increase in the brain may be. Many studies have suggested that spontaneous release of neurotrasmitters from axons occurs before the establishment of functional presynaptic terminals in developing neurons [10]. Spontaneous glutamate release from growing axons exerts a specific role prior to synapse formation to control dendritic arbor formation by signaling via “long-range” activation of N-Methyl-D-aspartate (NMDA) receptors on dendrites [23]. The activation of NMDA receptors could therefore conceivably provide one of the initial local Ca^2+^ raises to induce Cobl-mediated actin nucleation and filopodia initiation coupled to extensive branching on dendrites.

## Abbreviations

Arp2/3: Actin-related proteins 2/3
BAR: Bin–Amphiphysin–Rvs
CaM: Calmodulin
Cobl: Cordon-Bleu WH2 repeat protein
mDia1-3: Mammalian Diaphanous-related 1–3
NMDA: N-Methyl-D-aspartate
N-WASP: Neural Wiskott-Aldrich Syndrome Protein
PIP2: Phosphatidylinositol-4,5-bisphosphate
PIP3: Phosphatidylinositol-3,4,5-trisphosphate
WAVE-1: WASP-family Verprolin-homologous protein
WH2: WASP Homology 2
